# Delay to celiac disease diagnosis and its implications for health-related quality of life

**DOI:** 10.1186/1471-230X-11-118

**Published:** 2011-11-07

**Authors:** Fredrik Norström, Lars Lindholm, Olof Sandström, Katrina Nordyke, Anneli Ivarsson

**Affiliations:** 1 Department of Public Health and Clinical Medicine, Epidemiology and Global Health, Umeå University, Umeå, Sweden; 2 Department of Clinical Sciences, Pediatrics, Umeå University, Umeå, Sweden

## Abstract

**Background:**

To determine how the delay in diagnosing celiac disease (CD) has developed during recent decades and how this affects the burden of disease in terms of health-related quality of life (HRQoL), and also to consider differences with respect to sex and age.

**Methods:**

In collaboration with the Swedish Society for Coeliacs, a questionnaire was sent to 1,560 randomly selected members, divided in equal-sized age- and sex strata, and 1,031 (66%) responded. HRQoL was measured with the EQ-5D descriptive system and was then translated to quality-adjusted life year (QALY) scores. A general population survey was used as comparison.

**Results:**

The mean delay to diagnosis from the first symptoms was 9.7 years, and from the first doctor visit it was 5.8 years. The delay has been reduced over time for some age groups, but is still quite long. The mean QALY score during the year prior to initiated treatment was 0.66; it improved after diagnosis and treatment to 0.86, and was then better than that of a general population (0.79).

**Conclusions:**

The delay from first symptoms to CD diagnosis is unacceptably long for many persons. Untreated CD results in poor HRQoL, which improves to the level of the general population if diagnosed and treated. By shortening the diagnostic delay it is possible to reduce this unnecessary burden of disease. Increased awareness of CD as a common health problem is needed, and active case finding should be intensified. Mass screening for CD might be an option in the future.

## Background

Celiac disease (CD) is a permanent intolerance to gluten found in wheat, rye and barley. Gluten induces an autoimmune reaction in the small intestinal mucosa resulting in inflammation, villous atrophy and malabsorption. The only effective treatment is a gluten-free diet, which usually leads to healing of the intestinal mucosa and recovery from signs and symptoms [1]. Living with untreated CD is associated with a risk for extensive negative health consequences [2,3]. In some individuals the gastrointestinal symptoms are obvious, but frequently the symptoms are vague, which complicates the identification of CD cases. This has been shown both by an average delay of 11-13 years from symptoms to diagnosis [4-6], and through screening studies. Screening studies have shown five to 10 undiagnosed cases for every diagnosed case in some Western European countries [7], four of five Swedish adults with CD are undiagnosed [8], and two of three Swedish children with CD are undiagnosed [9]. In the latter study the prevalence was as high as 3%, indicating that CD is more common in some populations than the often mentioned 1%.

Health-related quality of life (HRQoL) as an aspect of living with CD has been studied frequently [4,5,10-17], although several questions still remain. The two generic utility-based instruments, Short Form 36 (SF-36) and EuroQol 5 Dimensions instrument (EQ-5D), are often used to measure HRQoL. SF-36 includes eight multi-item measures that represent different aspects of health [18]. EQ-5D includes both a descriptive system, comprised of five health-related dimensions (mobility, self-care, usual activities, pain/discomfort and anxiety/depression) divided into three levels of severity, and a visual analogue scale (VAS) for recording overall health [19]. Both instruments can be translated to a quality-adjusted life year (QALY) score, which enables comparisons between different diagnoses and with the general population. EQ-5D translation algorithms were first developed for the United States [20] and the United Kingdom (UK) populations [21], with the latter commonly used for the Swedish population [22]. For CD, SF-36 is still the most frequently used instrument [12,14-17], but EQ-5D has become increasingly popular [5,10].

Studies on treated CD adults have shown divergent results, with HRQoL either similar to [4,5,10] or worse than that of the general population [14,17]. Females with treated CD have been shown to experience worse HRQoL than males with treated CD [5,11-13]. One study using EQ-5D collected retrospective HRQoL data from members of a CD patient organization in the UK [5]. They concluded that the HRQoL before CD diagnosis is quantitatively similar to that of stroke patients, and that after initiated treatment it improved and was as good as that of the general population.

Despite many studies, unanswered questions remain, due in part to the small size of those studies. Previous studies have also lacked detailed analyses of various aspects due to the complex pattern regarding diagnostic delay and factors such as sex, age and time period for diagnosis. The aim of this study was to determine how the delay in diagnosing CD has developed during recent decades and how this affects the burden of disease in terms of HRQoL, and also to consider differences with respect to sex and age.

## Methods

### Study design

During 2009 we performed a cross-sectional questionnaire survey, of Swedish adults with CD that was approved by the Regional Ethical Review Board at Umeå University. The questionnaire was in Swedish and is accompanied to this article with an English translation [additional file 1]. For comparison, a questionnaire survey of the general adult population, "Health On Equal Terms" [23,24], was used. The latter was performed during 2006 and was approved by the ethical review board at the Swedish National Board of Health and Welfare.

### Study populations

We studied members of the Swedish Society for Coeliacs in 2009. The society is open to people with CD and other food intolerances and is the only one of its kind in the country. When joining the society, members self-report their food intolerance. At the time of the study, the society had 15,659 members reporting CD, 11,094 of whom were 20 years of age or older. Ludvigsson and colleagues defined 29,096 individuals diagnosed with CD in Sweden, 26,047 who were still alive, between 1969 and 2008, based on biopsy information from a computerized search of all regional pathology departments [25]. Thus, the society most likely represents about 60% of all persons with CD in Sweden. The general adult population study consisted of the four most northern Swedish counties, with a population of 677,777 persons 20 years of age or older.

### Subjects

In total, 1,560 adults with reported CD were invited to participate, with 65 males and females randomly selected from each five-year interval from 20 years of age and above (20-24, 25-29,..., 70-74, and 75 years or older). Out of 1,122 responders, 1,031 (66%) were eligible for the study. A reported CD diagnosis based on medical expertise was required for inclusion. As this information was lacking in the society's register, when respondents stated that they had CD, questionnaire information was used to assess how their CD was diagnosed (blood sample, biopsy, and/or diet change), and if a medical professional had recommended adherence to a gluten-free diet. Ninety-one members did not meet eligibility requirements, and they comprised three criteria groups; i) they did not have CD (n = 34), ii) CD diagnosis uncertain (n = 33), and iii) age and/or sex not consistent based on register information and questionnaire responses (n = 24). Those with uncertain CD were those reporting a self-diagnosis or that a gluten-free diet had not been recommended by a medical professional. Of eligible responders, 52% (n = 536) were females and the mean age was 52 years (Table 1).

**Table 1:** Characteristics of celiac disease subjects

	n	%	Mean	Median	Quartile	
					1st	3rd
Participants	1,031					
Males	495	48				
Females	536	52				
Age when responding (years)	1,031		52	53	36	67
Duration to diagnosis (years)						
First symptom	818		9.7	4	1	14
First doctor visit	853		5.8	1	0	8
Age at diagnosis for the whole group	945		39	41	27	53
0-19 years	162	17				
20-39 years	292	31				
40-59 years	353	37				
≥ 60 years	138	15				
Age at diagnosis in relation to period for diagnosis						
< 1980	67		17	2	1	37
1980-1989	162		27	32	1	45
1990-1999	272		39	43	30	52
2000-2004	221		46	47	33	59
2005-2009	444		45	46	31	59
Compliance with a gluten-free diet	1,025					
Strict	979	96				
Non-strict	46	4				
Household member with celiac disease	117	11				
Highest educational level	1,013					
Primary school	263	26				
Secondary school	414	41				
University degree	336	33				

In the general population survey 37,912 randomly selected persons were invited in strata of age, sex, county and municipality. In total, 27,809 (73%) responded, 25,797 of whom were 20 years or older [23]. Fifty-three percent (n = 13,781) were females and the mean age was 52 years.

### Questionnaires

Questionnaires were administered by the Swedish Society for Coeliacs (CD population) and by Statistics Sweden (general population), both distributed by the postal service to the persons' homes. To facilitate responding in both studies, a prepaid envelope was included and three reminders were sent out when needed. Questionnaire responses were scanned and thereafter checked for consistency.

The CD questionnaire included a section on delay to CD diagnosis with questions about the first appearance of symptoms possibly related to CD, the time of the first visit to a physician for those symptoms, and time of diagnosis. Information that could verify the CD diagnosis and self-reported degree of compliance with a gluten-free diet was also collected. We used the EQ-5D instrument to measure HRQoL. The respondent was asked about the year prior to initiated treatment for CD, referred to as *pre-treatment*, and about *today*, the time of responding to the questionnaire. The EQ-5D descriptive system was answered completely by 779 (76%) respondents and EQ VAS by 914 (89%) respondents.

In the general population, the EQ-5D descriptive system was completely responded to by 24,460 persons (95%) aged 20 years or more. EQ-VAS was not included in the questionnaire.

### Statistical analysis

Descriptive statistics were presented using frequency tables, cross-tabulations, and mean and median values. Means were compared with Student's t-test. Delay to diagnosis was estimated as the difference between the year of the first symptoms indicative of CD and the reported year of diagnosis. For a response of no symptoms before diagnosis, the delay was defined as 0 years. The delay from the first doctor visit was estimated in a similar way. Dependency between the time period for CD diagnosis and the delay to diagnosis was analyzed with the Cox proportional hazards model [26]. An exponential of the hazard ratio above 1 implies a shorter delay from first symptoms to diagnosis compared to the baseline, which was a diagnosis before 1980. The descriptive system was translated to a QALY score using UK weights [21]. Linear regression was used to study determinants for the QALY score *pre-treatment *and *today*. These analyses included sex, current age, delay from first symptoms indicative of CD to diagnosis (cutoff set to 2 years), and time period for diagnosis. Statistical significance was defined at the 5% level. Microsoft Access was used for data handling, while Stata 11.2 (StataCorp LP, College Station, TX) was used for statistical analysis.

## Results

### General characteristics

Of the 1,031 respondents, 52% were females. Mean and median ages at diagnosis were 39 and 42 years, respectively. Strict compliance with a gluten-free diet was reported by 979 (96%), and 117 (11%) reported a household member with CD (Table 1).

### Delay to CD diagnosis

The mean delay from the first symptoms indicative of CD to diagnosis was 9.7 years and the median delay was 4 years (quartiles 1-14 years). From the first visit to a doctor due to CD-related symptoms to diagnosis, the mean delay was 5.8 years and the median delay was 1 year (quartiles 0-8 years) (Table 1). Both males and females had a mean of at least 9 years from the first symptoms to diagnosis for each 5-year age group from 35-39 up to 65-69 years (data not shown). Excluding those below 20 years, no age group had a shorter mean delay than 6 years.

**Figure 1:**
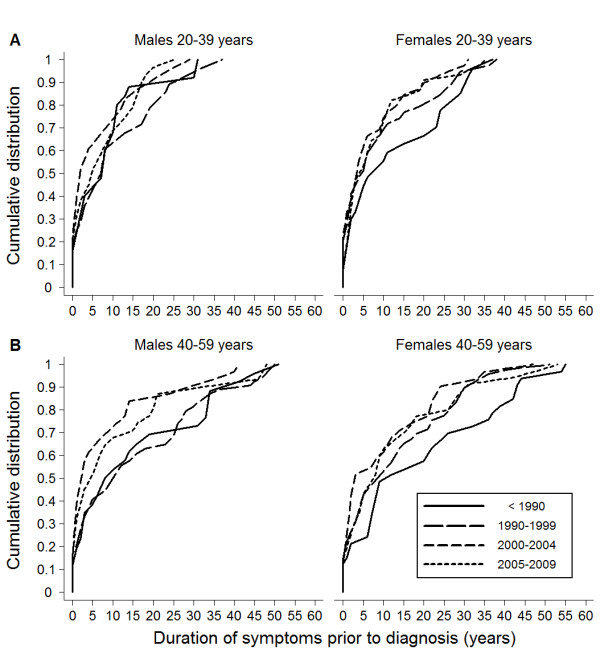
**Delay to celiac disease diagnosis from first symptoms indicative of the disease**. The delay is shown in groups formed by decade for diagnosis, sex and age at diagnosis (A) 20 to 39 years and (B) 40 to 59 years.

The median delay from symptoms to CD diagnosis has increased during recent decades, from 1 year for those diagnosed before 1980, to 5 years if diagnosed during the period 2005-2009 (data not shown). However, between these time periods the median age at diagnosis has increased from 2 years to 46 years (Table 1). Actually, the delay has decreased for some age groups in recent decades (Figure 1). This shift in delay is statistically significant for both males and females aged 20-39 years, and for females aged 40-59 years diagnosed during the period 2000-2004 as compared to before 1990 (Table 2). However, the delay is still considerable; for example, in 2005-2009, 56% of males and 47% of females aged 20-39 had a delay of at least 5 years (Figure 1). For the age groups 0-19 years and > 59 years at diagnosis, no statistically significant differences were observed with respect to delay to diagnosis when comparing time periods (data not shown).

**Table 2:** Relationship between time period for diagnosis and delay from first celiac disease symptoms to diagnosis

	**Age 20-39 years**				**Age 40-59 years**			
		
	**Males** **(n = 142)**		**Females** **(n = 148)**		**Males** **(n = 105)**		**Females (n = 150)**	
				
**Diagnosis year^a^**	**HR^b^**	**CI^c^**	**HR**	**CI**	**HR**	**CI**	**HR**	**CI**
				
< 1990	1	-	1	-	1	-	1	-
1990-1999	1.04	0.65-1.7	1.58	1.0-2.5*	0.81	0.47-1.4	1.29	0.78-2.1
2000-2004	1.71	1.0-2.9*	1.91	1.2-3.2*	1.27	0.71-2.3	1.70	1.0-2.8*
2005-2009	1.36	0.80-2.3	1.54	0.95-2.5	1.20	0.69-2.1	1.46	0.90-2.4

**^a ^**Cox proportional hazard using those diagnosed before 1990 as comparable group.

**^b ^**Exponential of the hazard rate.

^c ^Confidence interval.

* Significant difference at 5% level compared to diagnosis before 1990.

### Health-related quality of life

**Figure 2:**
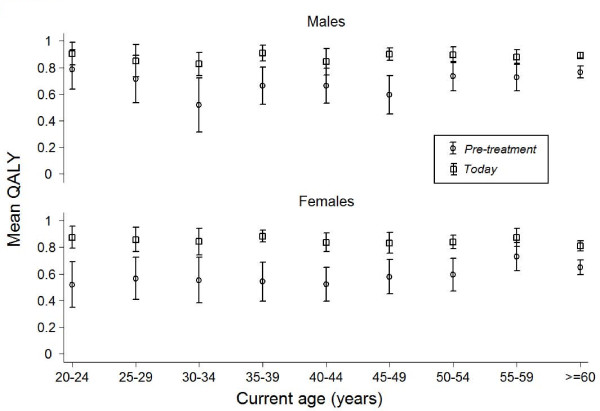
**Quality-adjusted life year (QALY) scores**. QALY scores *pre-treatment *for celiac disease and *today*, divided into current age and sex.

Within the EQ-5D descriptive system the dimension anxiety/depression differed most negatively for untreated CD patients when compared with the general population (Table 3). The mean QALY score for the CD population *pre-treatment *was 0.66, and it had increased to 0.86 *today *(p < 0.01) (Table 4). For the general population the score was 0.79, thus lower than for treated CD patients. Females reported a significantly lower QALY score than males, both for the CD population *pre-treatment *and *today *(Figure 2), as well as in the general population (p < 0.01). EQ VAS scores also improved after diagnosis (p < 0.01), with higher scores for CD males than for CD females (p < 0.01), both *pre-treatment *and *today *(Table 4).

**Table 3:** EQ-5D descriptive system

	Celiac disease (n = 779^a^)				General population (n = 24,460^a^)	
			
Dimensions	Pre-treatment	Today				
	**n**	**%**	**n**	**%**	**n**	**%**

Mobility						
No problems	720	92	737	95	20,899	85
Some problems	50	6.4	41	5	3,496	14
Severe problems	9	1.1	1	0.1	65	0.3
Self-care						
No problems	753	97	768	99	23,912	98
Some problems	14	1.8	7	0.9	442	1.8
Severe problems	12	1.5	4	0.5	106	0.4
Usual activities						
No problems	655	84	715	92	21,491	88
Some problems	95	12	54	6.9	2,529	10
Severe problems	29	3.7	10	1.3	440	1.8
Pain/discomfort						
No problems	291	37	481	62	9,484	39
Some problems	319	41	278	36	13,658	56
Severe problems	169	22	20	2.6	1,318	5.4
Anxiety/depression						
No problems	369	47	546	70	17,045	70
Some problems	314	40	222	28	6,898	28
Severe problems	96	12	11	1.4	517	2.1

^a ^Responded to all dimensions both *pre-treatment *and *today*.

**Table 4:** Quality-adjusted life year (QALY) scores and EQ-VAS

Celiac disease^a^											General population^a^		
		
	QALY^b^					EQ-VAS							
			
		Pre-treatment		Today		Pre-treatment		Today			QALY		
	**n**	**Mean**	**SD^c^**	**Mean**	**SD**	**n**	**Mean**	**SD**	**Mean**	**SD**	**n**	**Mean**	**SD**

Males	393	0.71	0.34	0.88	0.18	437	52	27	83	15	11,428	0.81	0.21
Females	386	0.60	0.37	0.84	0.21	477	44	25	80	17	13,032	0.77	0.23
All	779	0.66	0.36	0.86	0.19	914	48	27	81	16	24,460	0.79	0.22

^a ^Responded to all dimensions both *pre-treatment *and *today*.

^b ^QALY scores were calculated from responses to the EQ-5D descriptive system.

^c ^Standard deviation.

### Factors related to health-related quality of life

The QALY scores were significantly lower for CD females compared to CD males, both *pre-treatment *and *today *(Table 5). A lower QALY score *pre-treatment *was also associated with a younger current age, a long delay between first symptoms and diagnosis (more than 2 years), and being diagnosed before 1990 as compared to being diagnosed more recently (1990-2004) (Table 5).

**Table 5:** Factors related to health-related quality of life (HRQoL) *pre-treatment *for celiac disease (CD) and *today*

		**Pre-treatment**		**Today**
		
**Factor^a^**	**coeff.**	**p**	**coeff.**	**p**
		
Sex^**b**^	-0.10	< 0.001	-0.046	0.003
Current age (years)	0.0039	< 0.001	-0.0003	0.513
Long delay^**c**^	-0.14	< 0.001	-0.013	0.397
Diagnosed 1990-1999**^d^**	0.11	0.008	0.002	0.916
2000-2004**^d^**	0.13	0.002	-0.0008	0.971
2005-2009**^d^**	0.06	0.142	-0.011	0.615
Constant	0.49	< 0.001	0.91	< 0.001

**^a ^**Linear regression analysis was performed to predict HRQoL, measured with quality-adjusted life year (QALY) scores, including those who responded to all five dimensions of EQ-5D *pre-treatment *and *today *(n = 624).

^b ^Males = 0 and females = 1.

^c ^Delay to diagnosis from first symptoms indicative of CD > 2 years.

^d ^Comparing with those diagnosed before 1980.

## Discussion

CD patients experienced poor HRQoL *pre-treatment*, which improved significantly after diagnosis and treatment. Many experienced a long delay to diagnosis, both from the first symptoms and from the first visit to a doctor. It is promising that patients diagnosed after 2004 reported better HRQoL than those diagnosed earlier and that the delay in diagnosis has decreased during recent decades; however, many still experience too long a delay. There has been a pronounced increase in median age at diagnosis, from 2 to 46 years in recent decades, illustrating that CD has changed from presenting as a childhood disease to being a disease affecting all ages.

This is a large study with a high response rate. A strength is that we used a validated HRQoL instrument and compared HRQoL for the CD population with that of a general population. The approach of asking CD respondents about HRQoL both before and after initiated CD treatment has only been tried once before [5]. Our study is unique in that results for males and females are presented separately, and there is a more thorough analysis of factors that affect HRQoL. Further, we add results concerning how diagnostic delay has changed over time, also taking age and sex into consideration.

We believe that our findings are of great value for an increased understanding of how CD and its diagnostics affect people, despite some potential biases. We cannot be sure that our results are representative for the whole Swedish CD population. However, even if the results are only valid for responders (66% of invited) to the questionnaire within our study population (members of the Swedish Society for Coeliacs which correspond to about 60% of Swedish adults with CD), they nevertheless show an experienced burden for a sample representing about 40% of Swedish adults with CD, irrespective of age. The general population in our study is limited to northern Sweden while the CD population comes from the entire country. However, regarding HRQoL, there was no statistical difference between persons with CD from the northern part of the country and the rest of Sweden. A recall bias might appear as some responders had their CD diagnosis long ago. This might cause both a low estimate of HRQoL *pre-treatment*, as well as an overestimate of the delay from first symptoms indicative of CD to CD diagnosis. However, the usual recall pattern is to report fewer episodes of ill health, and therefore previous health problems might instead be underestimated.

A long diagnostic delay has been shown earlier in several studies [4-6]. However, awareness of CD has increased in recent decades and serological testing has been introduced and improved. Similar to findings in our study, a shortened diagnostic delay over time was found in the UK [5], but was not observed in Canada [4]. Neither study considered increasing age at diagnosis, a factor that might affect their results as well.

The positive effects of diagnosis and treatment we report here are also supported by others [5,10]. Our results confirm that CD females experience a lower HRQoL than males [13]. The difference is even larger before initiated treatment, which indicates that living with untreated CD might be a greater burden for females. It is also interesting that treated CD patients reported a better HRQoL than the general population. This might be a result of an altered frame of reference, after experiencing poorer health, or due to adapting to a healthier life style after being diagnosed with a chronic disorder. Our study subjects reported an unexpectedly high rate of compliance with a gluten-free diet (96%), and it has been shown that better compliance is related to a better HRQoL [11,17,27].

Despite increased awareness in society and in health care, many CD cases will be missed due to vague symptoms. Mass screening for CD has been raised as a possible option [28]. CD mass screening fulfils most of the listed criteria for a medical mass screening adapted by WHO from Wilson and Jungner [29]. However, knowledge concerning the natural history of CD and the cost-effectiveness of a CD mass screening is lacking [28,30,31]. There are few health economic evaluation studies of a CD mass screening. Existing studies indicate that a screening might be cost-effective [32,33], and that parents' willingness to pay for a CD mass screening on average is greater than the screening cost [34]. It was recently estimated in the United States that the medical cost for clinically detected CD patients is reduced by $1764 the year following diagnosis as compared to the average cost during the preceding years [35]. The fact that both cost savings and long-term health complications might differ between clinically diagnosed patients and screening detected CD must be taken into account, and there is a need for studies on this particular group [36,37].

## Conclusion

Our study shows that for many individuals there is an unacceptably long delay from the first symptoms to CD diagnosis. Untreated CD results in poor HRQoL that is improved to the level of the general population if diagnosed and treated. By shortening the diagnostic delay it is possible to reduce this unnecessary burden of disease. Increased awareness of CD as a common health problem is needed, and active case finding should be intensified. Mass screening for CD might be an option in the future.

## Competing interests

The authors declare that they have no competing interests.

## Authors' contributions

Study design by FN, AI, LL, and KN. FN coordinated data acquisition. FN performed the analyses and the interpretation in collaboration with AI, LL, and OS. FN drafted the paper and all co-authors contributed actively. All authors read and approved the final manuscript.

## Pre-publication history

The pre-publication history for this paper can be accessed here:

http://www.biomedcentral.com/1471-230X/11/118/prepub

## Supplementary Material

Additional file 1 **Questionnaire-To you, a member of the Swedish Society for Coeliacs**. Your experience is important!. Click here for file
