# Supplementary material for: Delay to celiac disease diagnosis and its implications for health-related quality of life
Source: BMC Gastroenterol. 2011 Nov 7;11:118. doi: 10.1186/1471-230X-11-118 (PMC3233515; doi:10.1186/1471-230X-11-118)
Supplement: Additional file 1 — Questionnaire-To you, a member of the Swedish Society for Coeliacs. Your experience is important!. [file 1471-230X-11-118-S1.PDF]

To you, a member of the Swedish Society for Coeliacs

Your experience is important!

To you, a member of the Swedish Society for Coeliacs,

We hope that you will spend some time helping us!

We want to increase the knowledge of what it is like to live with celiac disease, both before and after the disease has been confirmed, to find ways to improve the support from both the Society for Coeliacs and within health care. More information is given on the society's homepage (www.celiaki.se).

If you do not have celiac disease we still want you to respond to the first questions. If you have celiac disease, we want you to respond to all questions. *Your responses can not be given by someone else!* Send the questionnaire in the enclosed envelope (no stamps required). If you have misplaced the envelope, please use the address on the back of the questionnaire.

Please contact us if you have questions or concerns! You can reach us at the Swedish Society of Coeliacs (tel. 08-730 05 01), or through email (enkat@celiaki.se).

THANKS IN ADVANCE!
The Swedish Society for Coeliacs and Umeå University

---

1. Name ....

Sex
Man................ ☐
Woman .... ☐

Personal ID [| | | | |] - [| | |]

Street address ....

Postcode and town ....

Telephone ....

Email ....

2. Do you have celiac disease?

Yes .... ☐ => *Please* respond to all questions in the questionnaire.

No......... ☐ => If you do <u>not</u> have celiac disease, thanks for your participation!
Please return the questionnaire in enclosed envelope.

3. When was your celiac disease diagnosed? ☐☐☐☐ ☐☐

year *month*

4. Before your celiac disease was diagnosed, did you have trouble that might have been caused by untreated celiac disease?

Later in the questionnaire there are questions about symptoms you had before diagnosis.

Yes .... ☐ => What year did your troubles start? ☐☐☐☐ *year*

No......... ☐

5. Before your celiac disease was diagnosed, did you meet a doctor for troubles that might have been caused by untreated celiac disease?

Yes .... ☐ => What year did you meet a doctor for the first time? ☐☐☐☐ *year*

No......... ☐

6. How did you discover your celiac disease?

Tick one or more alternative(s)

Symptoms.. ☐

Test done because of another disease................ ☐ => What disease? ....

Test due to a family member with celiac disease . ☐

Self test... ☐

Other .... ☐ => How? ....

....

7. How was your celiac disease diagnosed?

Tick one or more alternative(s)

Blood test.. ☐

Biopsy from the small intestinal mucosa
Sometimes performed by gastroscopy .... ☐

Changed to a diet without gluten and felt better ... ☐

8. Has a health care professional recommended you to follow a gluten-free diet?

Yes .... ☐

No.................. ☐

9. Are you trying to follow a gluten-free diet?

Yes .... ☐

No.................. ☐

10. How strictly do you follow the gluten-free diet?

Always gluten-free .... ☐ *Always gluten-free or possibly a few unintentional mistakes*

Often gluten-free............... ☐

Sometimes gluten-free ..... ☐

Never gluten-free.............. ☐

11. After you started with the gluten-free diet; how did you experience the change in food expenses?

My food expenses have decreased .... ☐

My food expenses have increased .... ☐

My food expenses have increased a lot .... ☐

My food expenses have not been affected .... ☐

Do not know if my food expenses have been affected .. ☐

12. Are there others in your household with celiac disease?

Yes .... ☐

No .... ☐

13. Have you, or have you had, any of the following diseases?
If disease, please fill in when you were diagnosed.

	No	Yes		Year	Month
Lactose intolerance	☐	☐	=>		
Cow's-milk protein allergy....................................	☐	☐	=>		
Any other food intolerance...................................	☐	☐	=>		
Anaemia	☐	☐	=>		
Iron- or vitamin deficiency....................................	☐	☐	=>		
Diabetes -*Insulin treated*	☐	☐	=>		
-*Not insulin treated*	☐	☐	=>		
Osteoporosis	☐	☐	=>		
Rheumatic disease	☐	☐	=>		
Thyroid disease, e.g. goitre, hypothyroidism	☐	☐	=>		
Depigmentation of patches of skin (vitiligo)	☐	☐	=>		
Patches of hair loss (alopecia areata)	☐	☐	=>		
Dermatitis herpetiformis (rare skin disease)	☐	☐	=>		
Depression	☐	☐	=>		
Ataxia (problem with muscle coordination)	☐	☐	=>		
Inflammatory bowel disease,<br>e.g. ulcerative colitis, Crohn's disease..................	☐	☐	=>		
Any other disease(s), which?					

** Specify month by numbers, e.g. May=05*

14. How did you feel during the year prior to when your celiac disease was confirmed?

Excellent ☐ Very good ☐ Good ☐ Fairly good ☐ Bad ☐

15. Did you have any of the following symptoms during the year prior to when your celiac disease was diagnosed?
Tick one box in each row.

Symptom	Never	Seldom	Sometimes	Often	Always
Fatigue	☐	☐	☐	☐	☐
Abdominal pain.............................	☐	☐	☐	☐	☐
Upset stomach..............................	☐	☐	☐	☐	☐
Flatulence	☐	☐	☐	☐	☐
Hard stool	☐	☐	☐	☐	☐
Soft stool.......................................	☐	☐	☐	☐	☐
Heartburn......................................	☐	☐	☐	☐	☐
Nausea	☐	☐	☐	☐	☐
Vomiting..	☐	☐	☐	☐	☐
Weight loss	☐	☐	☐	☐	☐
Skin rash	☐	☐	☐	☐	☐
Mouth ulcer...................................	☐	☐	☐	☐	☐
Mood swings.................................	☐	☐	☐	☐	☐
Depression	☐	☐	☐	☐	☐
Headache	☐	☐	☐	☐	☐
Joint pain	☐	☐	☐	☐	☐
Body pain......................................	☐	☐	☐	☐	☐
Hair loss..	☐	☐	☐	☐	☐
Any other symptoms, specify					
	☐	☐	☐	☐	☐
	☐	☐	☐	☐	☐
	☐	☐	☐	☐	☐
	☐	☐	☐	☐	☐

16. How did you feel during the <u>previous year</u>?

Excellent ☐ Very good ☐ Good ☐ Fairly good ☐ Bad ☐

17. Did you have any of the following symptoms during the <u>previous year</u>?
Tick one box in each row

Symptom	Never	Seldom	Sometimes	Often	Always
Fatigue	☐	☐	☐	☐	☐
Abdominal pain.............................	☐	☐	☐	☐	☐
Upset stomach..............................	☐	☐	☐	☐	☐
Flatulence.....................................	☐	☐	☐	☐	☐
Hard stool	☐	☐	☐	☐	☐
Soft stool.......................................	☐	☐	☐	☐	☐
Heartburn......................................	☐	☐	☐	☐	☐
Nausea	☐	☐	☐	☐	☐
Vomiting..	☐	☐	☐	☐	☐
Weight loss	☐	☐	☐	☐	☐
Skin rash	☐	☐	☐	☐	☐
Mouth ulcer...................................	☐	☐	☐	☐	☐
Mood swings.................................	☐	☐	☐	☐	☐
Depression	☐	☐	☐	☐	☐
Headache	☐	☐	☐	☐	☐
Joint pain	☐	☐	☐	☐	☐
Body pain......................................	☐	☐	☐	☐	☐
Hair loss..	☐	☐	☐	☐	☐
Any other symptoms, specify					
	☐	☐	☐	☐	☐
	☐	☐	☐	☐	☐
	☐	☐	☐	☐	☐
	☐	☐	☐	☐	☐

18. By placing a tick in one box in each group below, please indicate which statements best describe your own health state <u>during the year prior</u> to when your celiac disease was diagnosed and <u>today.</u>

Tick in only one box for each group.

**Mobility**	*Year prior*	*Today*
I have no problems in walking about	☐	☐
I have some problems in walking about	☐	☐
I am confined to bed	☐	☐
**Self-care**		
I have no problems with self-care	☐	☐
I have some problems with washing or dressing myself	☐	☐
I am unable to wash or dress myself	☐	☐
**Usual activities** *(e. g. work, study, housework, family or leisure activities)*		
I have no problems with performing my usual activities	☐	☐
I have some problems with performing my usual activities	☐	☐
I am unable to perform my usual activities	☐	☐
**Pain/discomfort**		
I have no pain or discomfort	☐	☐
I have moderate pain or discomfort	☐	☐
I have extreme pain or discomfort	☐	☐
**Anxiety/depression**		
I am not anxious or depressed	☐	☐
I am to some extent anxious or depressed	☐	☐
I am to high extent anxious or depressed	☐	☐

Check that you ticked the boxes that best describe your health state, as you interpret it.

Health state
year prior
to diagnosis

19. To help people say how good or bad a health state is, we have drawn a thermometer-like scale on which the best state you can imagine is marked 100 and the worst health status you can imagine is marked 0.

Health state
today

Best imaginable health state

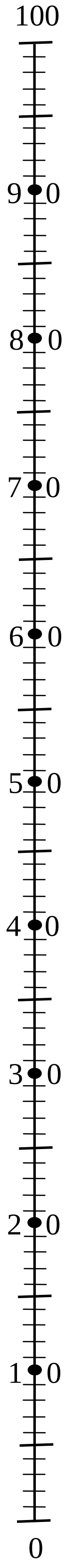

Worst imaginable health state

We would like you to indicate on **the scale on the left** how good or bad your health was, in your own opinion, **the year prior to your celiac disease was diagnosed**.

Please do this by drawing a **single line** from the grey box below to the point on the scale that indicates your previous health state.

We would like you to indicate on **the scale on the right** how good or bad your health is, in your own opinion, **today**.

Please do this by drawing a **single line** from the grey box below to the point on the scale that indicates your previous health state.

Health state year prior to diagnosis

Current health state

Best imaginable health state

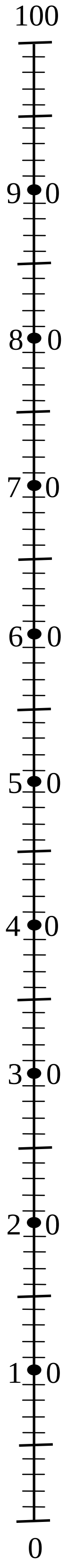

Worst imaginable health state

□□□ □□□

Do not write in these boxes!

20. Are there drugs that you have stopped using after your celiac disease was diagnosed?

No................. ☐

Yes .... ☐ => Specify which

....

....

21. About your weight

	Year prior to diagnosis	Today
Very underweight................	☐	☐
Somewhat underweight.......	☐	☐
Normal weight.....................	☐	☐
Somewhat overweight	☐	☐
Very overweight..................	☐	☐

22. Think of the <u>year prior</u> to when your celiac disease was diagnosed.

a) How often did you visit health care professionals?
e.g. doctor, district nurse, physiotherapist

☐☐ *Number of times*

b) How many days were you hospitalised?

☐☐ *Number of days*

c) How often did you miss working days, school days and similar?

☐☐ *Number of days*

23. Think of your situation <u>during last year</u>.

a) How often did you visit health care professionals?
e.g. doctor, district nurse, physiotherapist

☐☐ *Number of times*

b) How many days were you hospitalised?

☐☐ *Number of days*

c) How often did you miss working days, school days and similar?

☐☐ *Number of days*

24. What is your highest level of education?

Not finished 9 years of school..	☐
Finished 9 years of school (corresponding to Swedish elementary school) ...	☐
Finished 12 years of school (corresponding to Swedish high school)	☐
At least one year of education after high school	☐
Degree from university or college	☐

25. What is your employment status?

Employed, employee	☐
Employed, own business	☐
Studying..	☐
Job seeker, <u>more</u> than 6 months	☐
Job seeker, <u>less</u> than 6 months	☐
Work from home	☐
Parental leave...	☐
Retired, prematurely or disability-related	☐
Retired..	☐

26. What occupation do you have?

If you do not work at the moment, then specify previous occupation. Use a job title that as well as possible describes the occupation, e.g. school teacher or carpenter and <u>if willing describe briefly your job assignments.</u> For those with own business, we would also like to know number of employees who are in the company.

____

____

____

____

____

____

☐☐

Do not write in these boxes!

27. What channels do you use in order to achieve information about celiac disease and to communicate with us at the Swedish Society for Coeliacs?
Tick one or more alternative(s)

Bulletinen, our member's review .... ☐
www.celiaki.se, the association's web page .... ☐
Newsletter received by email .... ☐
Telephone times and email service from the association .... ☐
Your county and local society .... ☐

28. What information would you like from us?

____

____

29. Do you know that there is a research fund for celiac disease?

Yes .... ☐
No................. ☐

30. How often do you experience that your celiac disease limits your daily life?

Never ☐ Seldom ☐ Sometimes ☐ Often ☐ Always ☐

31. In what situation(s) do you feel limited because of your celiac disease?
Specify one or more alternative(s)

At the store .... ☐
At cafés, restaurants.. ☐
At school... ☐
Within health care .... ☐
At parties, social events.. ☐
At working place .... ☐
During travel .... ☐
During home visits to relatives or friends.............................. ☐
Other, specify
……………………………………………………………………… ☐
……………………………………………………………………… ☐

32. Do you know the symbol of the crossed-out wheat shaft?

Yes .... ☐

No........... ☐ => *What does the symbol mean?*

- Gluten-free...................... ☐
- Plant free .... ☐
- Without pesticide .... ☐
- Don’t know...................... ☐

33. Does the symbol of the crossed-out wheat shaft help you and others in your surroundings to find gluten-free products?

Yes .... ☐

No.................. ☐

Don’t know..... ☐

34. Is it important for you that the society works for a national economic grant (for both children and adults with celiac disease)?

Yes .... ☐

No.................. ☐

Don’t know..... ☐

35. Rank with numbers 1-8 what you think the society should work with.
1 the most important and 8 the least important.

☐ National economic grant

☐ Allergy marking at restaurants

☐ Better health care

☐ More allergy adjusted baking and food recipes

☐ Research

☐ Product range

☐ Increased cooperation with travel agencies

☐ More local meetings

What do you think?

Now you have filled in a long questionnaire,
but we would like you ask you for one more thing.

Your experience is important!

We would appreciate if you share with us how it is to live with celiac disease. Write it down in the way that suits you best, but try to cover the points below.

- Your way to diagnosis
  - Health-related, e.g. symptoms and diseases
  - Psychologically, e.g. worry, pressure and stress
  - Socially, e.g. family, friends and travels
  - Contacts with doctors and health care centres

- Life after diagnosis
  - Health-related, e.g. symptoms and diseases
  - Psychologically, e.g. worry, pressure and stress
  - Socially, e.g. family, friends and travels
  - Reflections about the future ….

____

____

____

____

____

____

____

____

____

____

____

____

____

____

____

____

____

____

Thanks for taking the time to fill in the questionnaire!

Your participation in the study will be of big value for the coeliac disease society's activity and will contribute to increased knowledge about celiac disease.

Please contact us if you have any views or thoughts about the study.

The Swedish Society for Coeliacs
Västra vägen 5B
169 61 Solna

Epidemiology and Global Health
Department of Public Health and Clinical Medicine
SE-901 87 Umeå
